# Primary pulmonary mesenchymal malignancy associated with paraneoplastic pemphigus: a case report

**DOI:** 10.3389/fmed.2026.1844671

**Published:** 2026-06-10

**Authors:** Yi Dong, Yongjie Guan, Shuang Feng, Xiaowei Wang, Xiyuan Ren, Ke Gao

**Affiliations:** 1 Department of Cardio-Thoracic Surgery, School of Clinical Medicine, North Sichuan Medical College, Nanchong, Sichuan, China; 2 West China School of Medicine, Sichuan University Affiliated Chengdu Second People’s Hospital, Chengdu Second People’s Hospital, Sichuan University, Chengdu, Sichuan, China; 3 Department of Cardio-Thoracic Surgery, West China School of Medicine, Sichuan University Affiliated Chengdu Second People’s Hospital, Chengdu Second People’s Hospital, Sichuan University, Chengdu, Sichuan, China; 4 Clinical Medicine, Chengdu Medical College, Chengdu, Sichuan, China

**Keywords:** lung neoplasms, lymphoproliferative disorders, multiorgan syndrome, paraneoplastic pemphigus, pulmonary sarcoma

## Abstract

Primary pulmonary inflammatory myofibroblastic tumor (IMT) associated with paraneoplastic pemphigus (PNP) is extremely rare. IMT is an intermediate (rarely metastasizing) mesenchymal neoplasm rather than a conventional sarcoma. We report a case of a 68-year-old female patient who initially presented with generalized skin rash, oral mucosal ulceration, and fever. Chest CT revealed a thoracic mass (initially difficult to determine whether mediastinal or pulmonary in origin). Subsequent thoracotomy and surgical resection with pathological examination confirmed an ALK-rearranged inflammatory myofibroblastic tumor (SQSTM1: ALK fusion) of pulmonary origin. Oral mucosal pathology, combined with clinical findings, supported a diagnosis of PNP. This case aims to provide reference for the diagnosis and treatment of patients with primary pulmonary IMT complicated by PNP. However, the follow-up period was short and systemic immunosuppressive therapy was not administered; this should be regarded as a cautionary note rather than a therapeutic recommendation.

## 1 Introduction

Paraneoplastic pemphigus/paraneoplastic autoimmune multiorgan syndrome (PNP/PAMS) is a rare autoimmune disease that is almost invariably associated with an underlying diagnosed or occult malignancy (1). PNP/PAMS is a highly lethal autoimmune blistering disease that occurs in patients with benign or malignant tumors, most commonly lymphoproliferative disorders. Both humoral and cell-mediated immunity are involved in its pathogenesis. Patients typically present with severe stomatitis and polymorphous skin lesions that are often refractory to treatment. Bronchiolitis obliterans (BO) is a common complication that contributes to the high mortality rate of PNP/PAMS (2).

Inflammatory myofibroblastic tumor (IMT) is an intermediate (rarely metastasizing) mesenchymal neoplasm with clonal origin, frequently harboring ALK gene rearrangements (3). Primary pulmonary IMT is uncommon in adults, accounting for less than 1% of all lung tumors (4–6). IMT must be distinguished from metastatic sarcoma to the lung, primary pulmonary sarcomatoid carcinoma, and diffuse malignant mesothelioma involving the lungs. Additionally, pulmonary malignant spindle cell lesions, primary chest wall or mediastinal tumors, and tumors that may be difficult to distinguish from pulmonary origin based on the degree of thoracic vertebral involvement must be considered (7).

To our knowledge, previously reported IMT-associated PNP cases have involved extrapulmonary sites, including the retroperitoneum, mediastinum, abdomen, and intercostal nerve (8, 9). Clinical outcomes in these cases have been highly dichotomous: complete tumor resection achieved long-term remission or cure, whereas the development of bronchiolitis obliterans (BO) was almost invariably fatal (10). This study reports what we believe to be one of the first documented cases of primary pulmonary IMT associated with paraneoplastic pemphigus, characterized by SQSTM1: ALK fusion, adding novelty in both anatomical origin and molecular features.

## 2 Case report

A 68-year-old female patient was admitted due to lip ulcerative lesions, widespread skin rash, and fever. The mucocutaneous symptoms had begun approximately 7 months prior to admission, initially with lip ulceration that was not taken seriously. Four months before admission, she visited a local hospital where chest CT revealed a thoracic mass (difficult to determine whether pulmonary or mediastinal in origin). Seven days before admission, widespread rash and fever developed, prompting presentation to our hospital.

**Figure 1:**
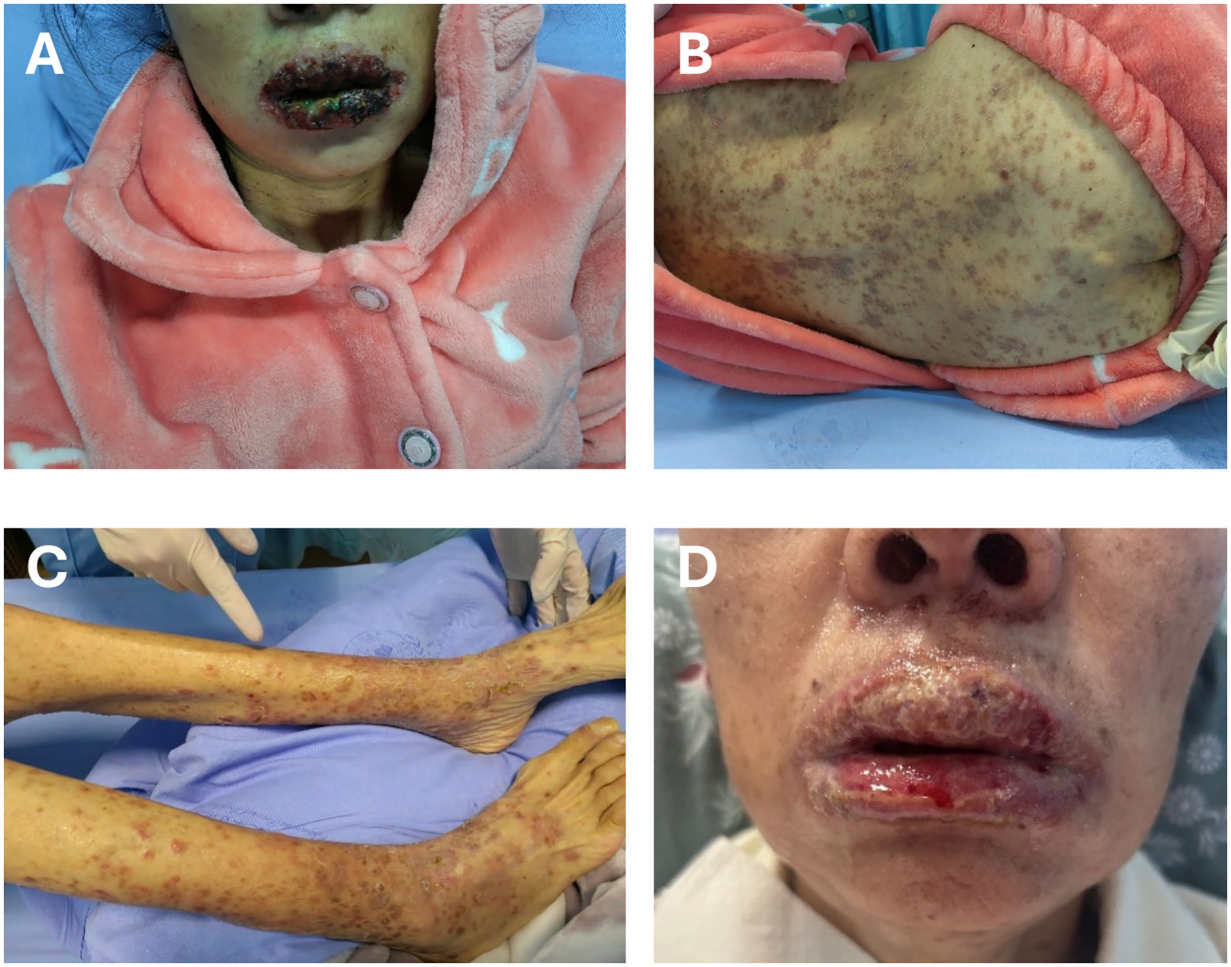
Clinical manifestations of mucocutaneous lesions. **(A)** Preoperative lip ulcerative lesions; **(B)** widespread back rash; **(C)** lower extremities rash; **(D)** lip recovery status during postoperative rehabilitation.

The rash was distributed throughout the body, including the limbs, palms and fingers, chest and back, and vulva, without symptoms of itching or pain (partial rash manifestations shown in Figures 1A–C). Due to inability to eat because of oral involvement, the patient had poor nutritional status and was emaciated.

**Figure 2:**
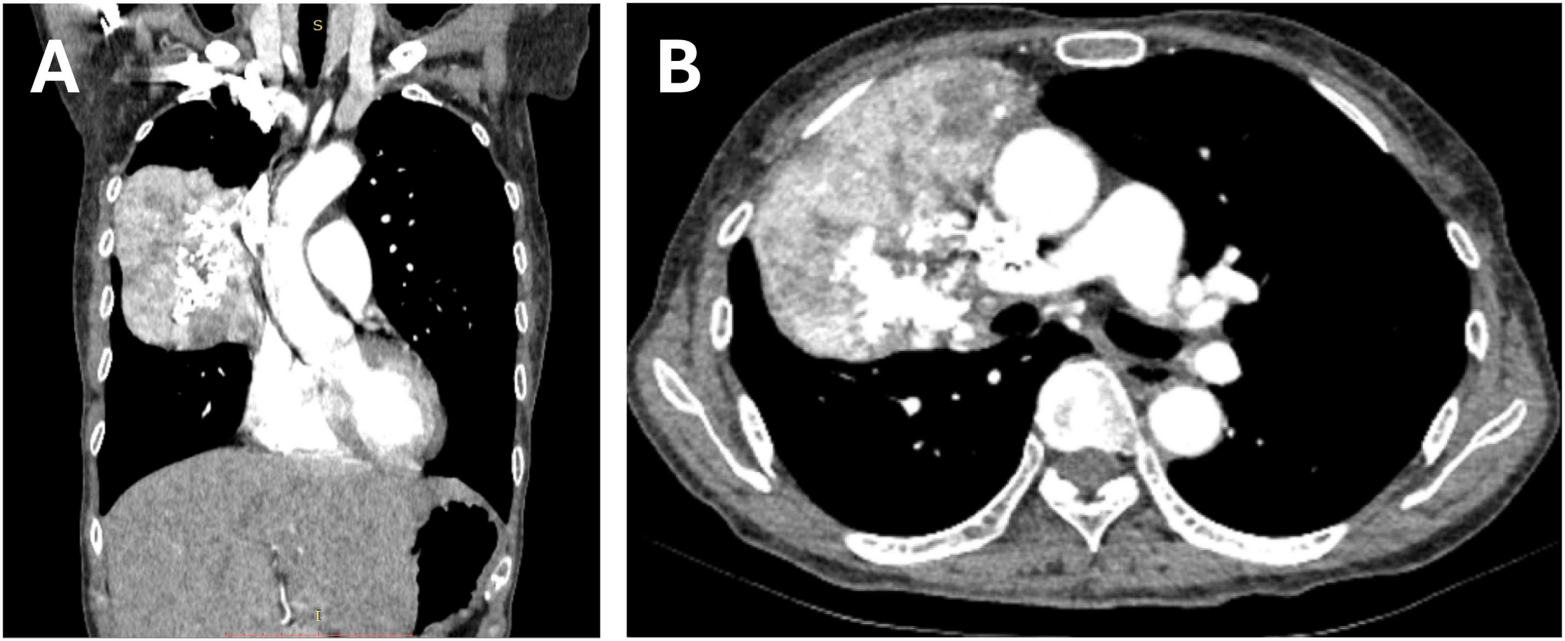
Chest CT imaging. **(A)** Coronal CT image; **(B)** cross-sectional CT image of the tumor.

Upon admission, chest CT revealed a thoracic mass (Figure 2): a soft tissue density mass in the right thoracic-mediastinal region, measuring approximately 104 mm × 77 mm, with marked heterogeneous progressive enhancement. The mass contained multiple dense shadows and poorly enhanced low-density areas. The tumor surrounded or was closely adjacent to the pulmonary arteries and veins, with tortuous and dilated vessels visible at the tumor margin and poorly defined borders with the adjacent pleura. The adjacent right upper and middle lobe bronchi were compressed and narrowed, with corresponding atelectasis of the lung tissue. No enlarged mediastinal lymph nodes were observed. These imaging features—large size, heterogeneous enhancement, presence of calcification, and absence of lymphadenopathy—are more consistent with a mesenchymal neoplasm such as IMT than with primary lung carcinoma, which more commonly presents with irregular margins, necrosis, and lymph node metastasis (11).

Interventional puncture of the pulmonary mass yielded a small sample, with pathology indicating a mesenchymal-derived malignant tumor; however, further subtyping was not possible due to the limited sample size. Fiberoptic bronchoscopy revealed a small amount of white viscous secretions in the right bronchus, with normal openings of each lobe and segment and no neoplastic lesions visible.

**Figure 3:**
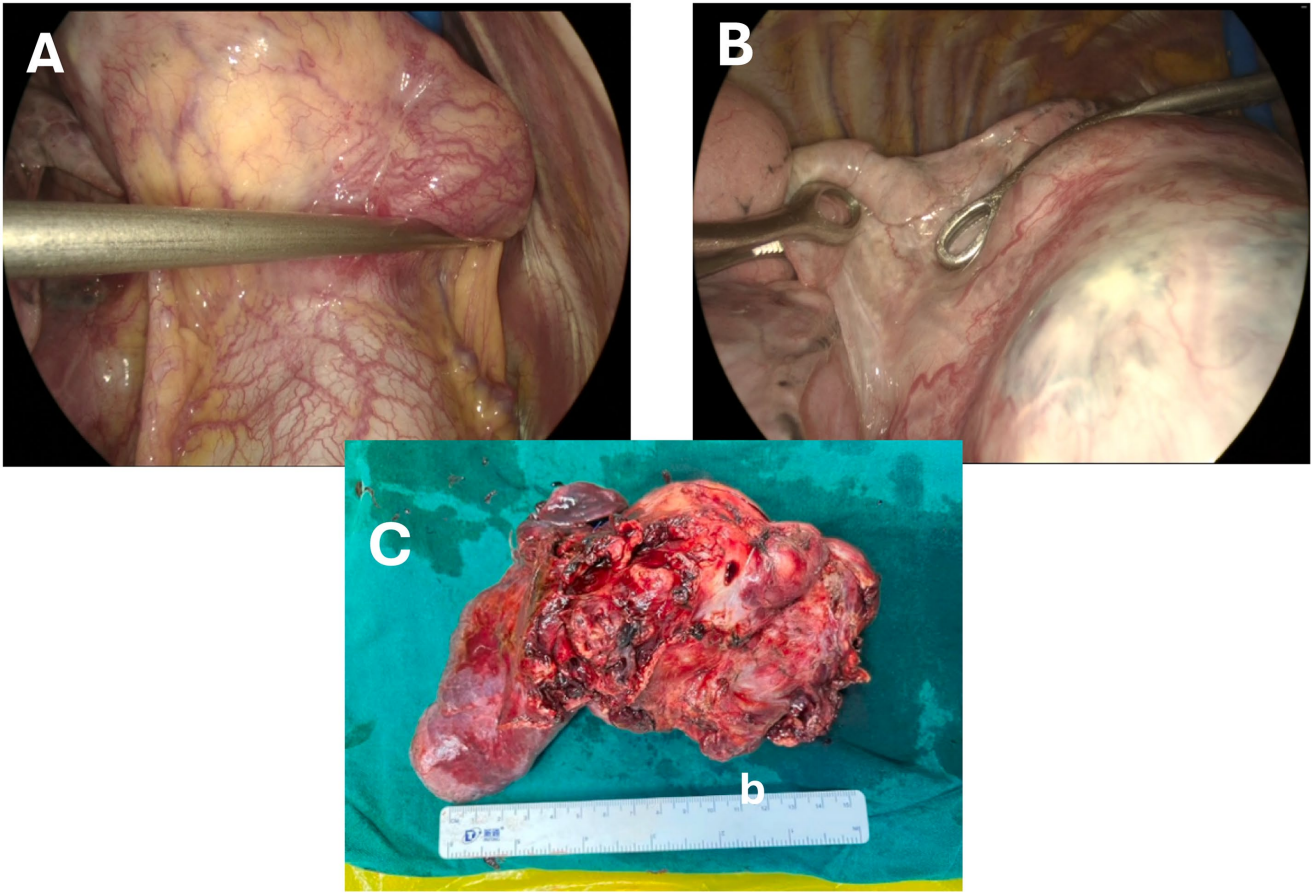
Intraoperative findings and resected specimen. **(A)** Intraoperative finding of the relationship between tumor and mediastinum (pericardium); **(B)** Intraoperative view showing tight adhesion between tumor and lung tissue; **(C)** Resected right upper and middle lobes with tumor tissue, a: pulmonary resection margin, b: mediastinal resection margin.

After excluding metastatic tumors through systemic imaging evaluation (no evidence of extrapulmonary primary soft tissue tumor or distant metastatic disease), radical surgical resection was performed. Given the large size of the tumor, thoracotomy was chosen. Intraoperative exploration revealed that the mass was predominantly located in the right upper and middle lobes of the lung, with infiltrative growth into the pulmonary parenchyma and secondary involvement of the pericardium (Figures 3A,B). Therefore, right upper and middle lobectomy with partial pericardial resection was performed. Partial mediastinal lymph node dissection was also performed, with no metastasis identified in any nodes. The mass measured approximately 13 cm × 10 cm × 6.5 cm, with partial calcification visible to the naked eye (Figure 3C). The surgery was completed successfully.

During the postoperative hospitalization following mass resection, the patient’s generalized skin rash and oral lesions gradually improved (Figure 1D). However, 2 weeks postoperatively, the patient suddenly developed decreased oxygen saturation to 40% of unknown cause while in the ward. She was subsequently transferred to the ICU for endotracheal intubation. After intubation, oxygen saturation did not immediately improve. Suctioning through the endotracheal tube yielded large amounts of thick sputum, after which oxygen saturation rapidly rose to 95%. During this episode, D-dimer was 1.62 μg/mL; pulmonary artery CTA excluded pulmonary embolism. Arterial blood gas analysis showed pH 7.18, pO₂ 41 mmHg, and pCO₂ 95 mmHg. The patient received oxygen therapy and acid–base correction in the ICU. After 2 days, vital signs gradually stabilized, and she was transferred back to the general ward. Due to the emergent nature of the event, high-resolution CT (HRCT), bronchoscopy, or pulmonary function tests were not performed. Therefore, bronchiolitis obliterans (BO) could not be confirmed or excluded. This clinical course suggests that acute airway obstruction due to mucus plugging may have been the primary contributing factor, although BO remains a suspected but unproven complication.

Final paraffin pathology reports were obtained 1 month postoperatively.

**Figure 4:**
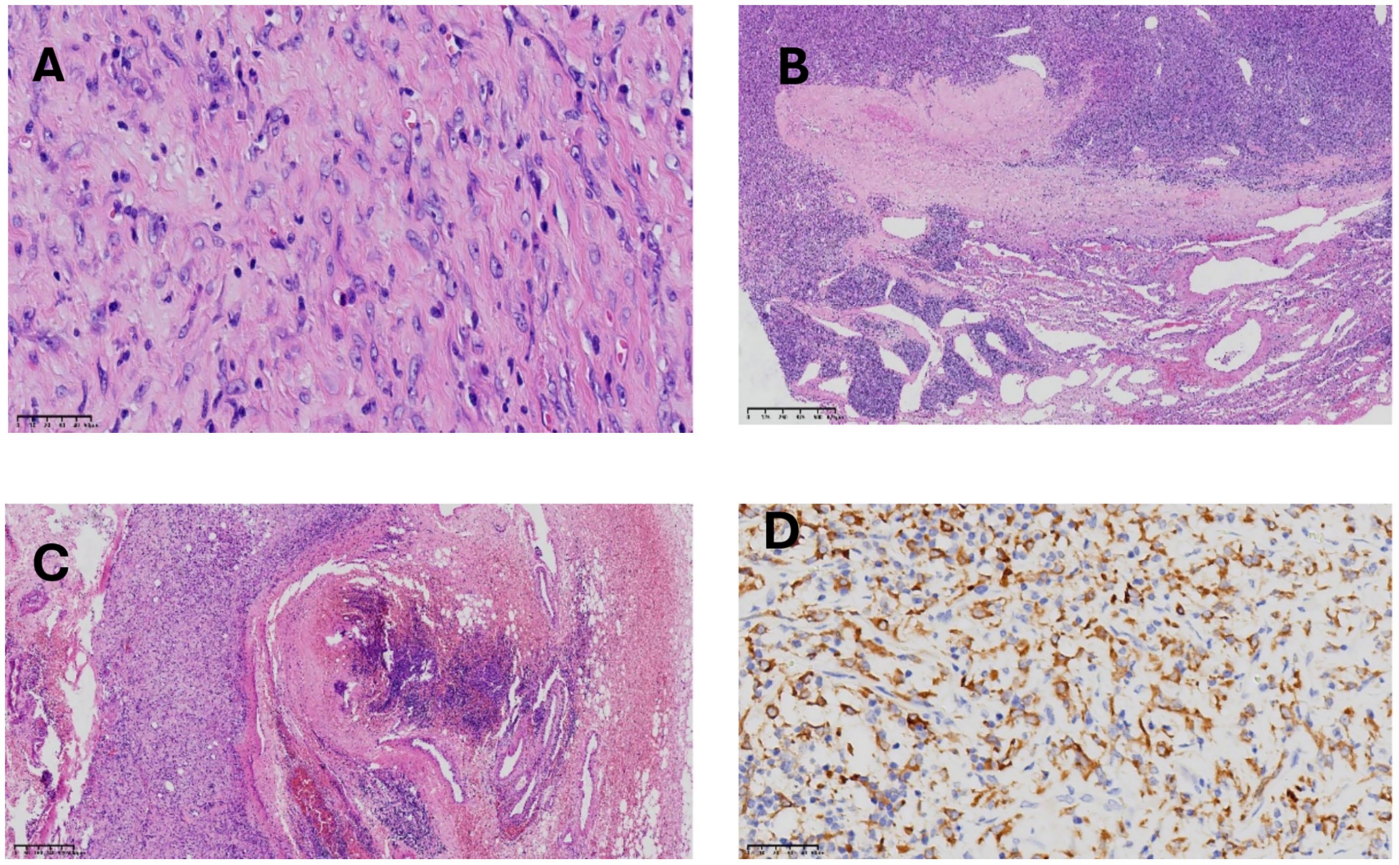
Histopathological and immunohistochemical findings. **(A)** Tumor section (40 × magnification); **(B)** tumor and lung tissue section (4 × magnification); **(C)** relationship between tumor and mediastinum (4 × magnification); **(D)** tumor characteristic immunohistochemistry ALK (4 × magnification); **(E)** peribronchiolar inflammation (10 × magnification); **(F)** lip ulcer section (20 × magnification).

The surgical specimen was sent for paraffin pathology (histopathological sections shown in Figures 4A–E). Histologically, IMT is characterized by spindle cell/myofibroblastic proliferation with a mixed inflammatory infiltrate including lymphocytes and plasma cells. Tumors typically exhibit variable cellularity and stromal patterns. Features suggestive of more aggressive biological behavior include increased mitotic activity, large tumor size, and infiltrative growth margins. In this case, the combination of spindle cell proliferation, inflammatory background, and ALK positivity supported the diagnosis of IMT (12). The tumor showed diffuse or nodular distribution, with some areas having stroma rich in thin-walled vessels; nodular areas showed obvious fibrosis and hyalinization. Tumor cells were epithelioid or spindle-shaped, with round or oval nuclei, vesicular chromatin, relatively prominent nucleoli, slightly eosinophilic cytoplasm, and 1–3 mitotic figures per 2 mm^2^. The stroma showed infiltration of numerous lymphocytes, plasma cells, and a few eosinophils, with multiple foci of atrophic lymphoid follicles visible. The tumor (size approximately 13 cm × 10 cm × 6.5 cm) had ill-defined margins, involving hilar lung tissue and mediastinal soft tissue, with partial ossification near the hilum and focal cystic degeneration.

The patient underwent genetic testing, which revealed SQSTM1: ALK fusion. Immunohistochemical results are summarized in Table 1.

**Table 1:** Key Immunohistochemical Results.

Marker	Result	Interpretation
ALK (clone 5A4)	Positive (cytoplasmic)	Characteristic of ALK-rearranged IMT
SMA	Positive	Myofibroblastic differentiation
SMARCA4/Brg1	Positive (not lost)	Excludes SMARCA4-deficient tumor
INI-1	Positive (not lost)	Excludes INI-1-deficient tumor
CD68	Negative (histiocytes positive)	Excludes histiocytic neoplasm
CD163	Negative (histiocytes positive)	Excludes histiocytic neoplasm
PAX-5	Negative (B cells positive)	Excludes lymphoid neoplasm
CD21	Negative	Excludes follicular dendritic cell tumor
CD23	Negative	Excludes follicular dendritic cell tumor
CXCL13	Negative	Excludes follicular dendritic cell tumor
CD1a	Negative	Excludes Langerhans cell histiocytosis
Langerin	Negative	Excludes Langerhans cell histiocytosis
Desmin	Negative	Excludes muscle differentiation
EMA	Negative	Excludes epithelial/carcinoma differentiation
MyoD1	Negative	Excludes rhabdomyosarcoma
Calponin	Negative	Excludes smooth muscle tumor
Dog-1	Negative	Excludes GIST
MUC4	Negative	Excludes low-grade fibromyxoid sarcoma
TFE3	Negative	Excludes TFE3-rearranged neoplasm
Ki-67	Positive (~10%)	Low-to-intermediate proliferative activity

Combining histological morphology, puncture biopsy, and subsequent detection results from the surgical specimen, the pathological diagnosis was inflammatory myofibroblastic tumor (IMT), but with potentially more aggressive biological behavior than classical IMT. The tumor nature was recommended to be classified as low-to-intermediate grade malignancy.

Oral mucosal ulcer tissue was sent for pathology (histopathological section shown in Figure 4F): mucosal erosion and ulceration with crusting, focally residual incomplete squamous epithelium, with a few acantholytic cells visible above the basal layer and numerous neutrophils exuding in the surrounding area; stroma showed abundant plasma cell infiltration, with occasional multinucleated giant cells focally. The microscopic appearance, combined with clinical findings, was consistent with paraneoplastic pemphigus (PNP). However, complete immunologic work-up—including direct and indirect immunofluorescence and ELISA panels for envoplakin, periplakin, desmoplakin, and BP230—was not performed. Therefore, the PNP diagnosis was based on clinical and histologic criteria, which may be less specific than the full immunologic gold standard.

According to existing evidence, PNP patients still require systemic immunosuppressive therapy postoperatively, and high-dose glucocorticoids remain first-line treatment for PNP (13). However, the patient recovered well during the postoperative hospitalization and had a strong desire for discharge. After fully considering the patient’s needs and evaluating her physiological status, systemic glucocorticoid therapy and immunosuppressive therapy were not administered. This deviation from standard PNP treatment reflects an individualized clinical decision made under real-world constraints rather than a recommended approach. The patient was instructed to follow up in the outpatient clinic 1 month after discharge and was subsequently discharged. Unfortunately, the patient did not return for follow-up at our institution.

## 3 Discussion

### 3.1 IMT epidemiology, pathology, and ALK-fusion biology

Inflammatory myofibroblastic tumor (IMT) is a mesenchymal tumor that may occur in soft tissues of almost all organs and may also occur in the lung or mediastinum. A substantial proportion of IMTs harbor ALK gene rearrangements, leading to constitutive activation of STAT3, PI3K/AKT, and MAPK signaling pathways, which promote tumor growth and may influence the tumor immune microenvironment (3). Immunohistochemical characteristics show considerable overlap between pulmonary and mediastinal locations and determining origin by immunophenotype is limited (14); therefore, anatomical localization of the tumor source is more important. In this case, intraoperative findings revealed that the tumor was predominantly located in the right upper and middle lobes of the lung, with infiltrative growth into the pulmonary parenchyma and secondary involvement of the pericardium and thymus, supporting a diagnosis of pulmonary-origin IMT.

IMT is relatively common in children, accounting for 20–50% of all primary pediatric lung tumors, but accounts for less than 1% of all adult lung tumors (4–6). Although IMT is generally considered a neoplasm of intermediate malignant potential, its clinical behavior is diverse and may include locally aggressive, recurrent, and metastatic disease. The diagnosis of IMT depends on histological examination. Tissue samples obtained by fine needle aspiration and true-cut biopsy are usually too small to make a reliable diagnosis (15). In this case, initial puncture only indicated a mesenchymal-derived malignant tumor; definitive diagnosis required paraffin pathology and genetic testing of the resected mass, a process that took nearly 1 month.

### 3.2 PNP/PAMS pathogenesis, prognosis, and link to BO

PNP/PAMS is associated with underlying tumors in almost all cases. Literature review has found that non-Hodgkin lymphoma is most common, followed by chronic lymphocytic leukemia (CLL) and Castleman’s disease (16). Sarcomas including reticulum cell sarcoma, liposarcoma, dendritic cell sarcoma, leiomyosarcoma, malignant schwannoma, and inflammatory myofibroblastic tumors account for 6.2% of PNP/PAMS cases (16). PNP/PAMS is characterized by the formation of autoantibodies against keratinocyte surface molecules, leading to acantholysis or loss of adhesion function between keratinocytes (17). This is also an extremely lethal disease, with reported mortality rates typically between 50 and 80%. The most common causes of death are related to infection, respiratory failure secondary to BO, PNP/PAMS itself, and the underlying tumor (10).

Epitope spreading and autoantibodies produced by underlying tumors are two mechanisms of autoantibody production in PNP/PAMS. Both humoral and cellular immunity play key roles in the pathogenesis of mucocutaneous lesions and bronchiolitis obliterans (BO) in PNP/PAMS (2). In this case, the presence of SQSTM1: ALK fusion suggests that tumor-associated antigenic stimulation may participate in immune activation. ALK may promote chronic immune activation through STAT3, PI3K/AKT, MAPK, and inflammation-related pathways (3). In ALK-rearranged IMT, persistent ALK fusion protein expression and the tumor inflammatory microenvironment may promote the extension of antitumor immunity toward autoimmune reactions against epithelial adhesion molecules. This represents a biologically plausible but speculative mechanism by which ALK might contribute to PNP pathogenesis; however, the specific role of ALK fusion in this process remains to be further studied.

### 3.3 Specific learning points from this case

#### 3.3.1 Diagnostic approach to thoracic mass in PNP

The patient presented with PNP as the primary clinical manifestation. Chest CT at our hospital showed a large mass without mediastinal lymph node enlargement, with osteoid calcification and necrosis within the mass—features suggestive of mesenchymal tumors. Literature has reported that on chest CT, larger lesion size, location near the lower lobe, parietal or hilar position, low mean attenuation density, presence of lobulated margins, calcification and fissure extension, and absence of emphysematous changes or distant metastasis in young patients are more indicative of pulmonary sarcoma than lung cancer (11). However, primary pulmonary mesenchymal neoplasms still lack characteristic clinical and imaging manifestations and are easily misdiagnosed (18). Imaging still cannot determine the type of mass; determination of the nature of thoracic masses is crucial for subsequent treatment. Only pathological biopsy can provide definitive diagnosis, so mass puncture biopsy was performed to clarify nature. After determining it was a mesenchymal-derived malignant tumor and excluding other metastatic conditions through systemic imaging, surgical resection was performed.

The diagnosis of primary pulmonary IMT in this case was established through comprehensive integration of imaging, intraoperative findings, histopathology, immunohistochemistry, and molecular testing. First, preoperative imaging demonstrated a thoracic mass closely related to the right upper and middle lobes, with no evidence of extrapulmonary primary soft tissue tumor or distant metastatic disease. Intraoperatively, the tumor was predominantly located in and infiltrating the right upper and middle lobes, with secondary involvement of adjacent mediastinal soft tissue and pericardium, supporting pulmonary rather than mediastinal or pleural origin. Second, given the absence of extrapulmonary primary lesions and no metastasis in mediastinal lymph nodes, metastatic sarcoma was considered unlikely. Third, the tumor lacked epithelial differentiation both morphologically and immunophenotypically, excluding pulmonary sarcomatoid carcinoma; epithelial markers such as EMA were negative, while cytoplasmic ALK and SMA were positive. Finally, the presence of SQSTM1: ALK fusion further supported the diagnosis of ALK-rearranged IMT. Collectively, these findings support a diagnosis of primary pulmonary IMT with locally infiltrative growth, rather than metastatic sarcoma, pulmonary sarcomatoid carcinoma, or primary mediastinal/pleural tumor.

#### 3.3.2 Perioperative respiratory risk

Considering that the most dangerous complication of PNP is bronchiolitis obliterans, and the patient experienced postoperative oxygen saturation decreases to 40%, BO was included in the differential diagnosis. The pathological feature of BO is patchy fibrosis beneath the epithelium of alveolar ducts, leading to near-complete or complete obliteration of the airway lumen (19). In this case, pathological sections showed peribronchiolar inflammatory changes (Figure 4E) but no obvious bronchiolar fibrosis. Nevertheless, this reminds us that respiratory management and monitoring are essential.

#### 3.3.3 Therapeutic considerations

Clinical management of PNP/PAMS is mostly based on case reports and small sample experiences. Existing evidence suggests that complete resection or effective control of the underlying tumor is a key step in improving immune abnormalities and relieving skin and mucosal damage, but most patients still require combined systemic immunosuppressive therapy. Based on high-dose glucocorticoids, immunosuppressants such as cyclophosphamide, azathioprine, or mycophenolate mofetil are often needed to enhance efficacy and reduce steroid dependence (13). Complete clearance of mucocutaneous lesions and normalization of serum antibodies may take months to years (20–22). Most PNP/PAMS patients with more aggressive underlying tumors will have progressive skin symptoms even after surgical resection (21).

Although systemic corticosteroids and immunosuppressants remain standard treatment for PNP, real-world management becomes challenging when patients decline immunosuppressive therapy due to concerns about adverse effects, infection risk, or long-term toxicity. In such situations, maximal tumor-directed therapy becomes particularly important because PNP is fundamentally driven by the underlying tumor. Where feasible, complete surgical resection remains the preferred strategy. In ALK-rearranged IMT, ALK-targeted therapy may represent a biologically plausible alternative to reduce persistent antigenic stimulation and tumor-associated immune activation. Several supportive strategies have been reported in the literature, including aggressive supportive care, intravenous immunoglobulin (IVIG), pulmonary monitoring, and bronchiolitis obliterans-directed therapies (such as inhaled corticosteroids, azithromycin, montelukast, and bronchodilator therapy), among which respiratory monitoring and multidisciplinary management are crucial (23).

### 3.4 Forward-looking therapeutic perspective and follow-up strategy

Current reviews indicate that ALK-positive IMTs, including those with SQSTM1: ALK fusion, may respond to ALK inhibitors, and these agents are increasingly used in unresectable, recurrent, or metastatic disease (3). Although this patient achieved complete surgical resection, ALK inhibitors might theoretically represent a biologically rational treatment option should recurrence occur.

Given the dual risk from IMT (recurrence) and PNP (BO, infection), optimal follow-up should include regular clinical review, periodic imaging surveillance, and pulmonary function assessment as indicated. Multidisciplinary management is essential, requiring coordination among thoracic surgery, dermatology, pathology, pulmonology, and rheumatology/immunology as appropriate, particularly regarding PNP management and BO monitoring.

### 3.5 Clinical lessons

1. *Value of considering IMT in large calcified thoracic masses*: Large, calcified thoracic masses with heterogeneous enhancement and absent lymphadenopathy should raise suspicion for mesenchymal neoplasms such as IMT, particularly in the context of paraneoplastic syndromes.
2. *Need for early PNP recognition*: Severe mucocutaneous lesions with underlying malignancy should prompt consideration of PNP, even when immunologic testing is unavailable.
3. *Importance of respiratory monitoring and BO surveillance*: Given the high mortality associated with BO in PNP, vigilant postoperative respiratory monitoring is essential, even when BO cannot be confirmed.
4. *Individualized therapeutic decision-making*: When patients refuse standard immunosuppressive therapy, maximal tumor-directed therapy and close multidisciplinary follow-up become paramount, though this should not be interpreted as a recommended standard strategy.

## 4 Conclusion

We report a rare case of primary pulmonary inflammatory myofibroblastic tumor (ALK-rearranged, SQSTM1: ALK fusion) associated with paraneoplastic pemphigus. Due to the lack of evidence-based guidelines for the management of PNP/PAMS complicated by pulmonary IMT and the absence of randomized clinical trials to guide treatment, management relies mainly on case reports and case series. Thoracic surgeons need professional understanding of clinical knowledge to initiate appropriate diagnostic and treatment measures for early diagnosis and management. However, conclusions about prognosis or optimal management cannot be generalized from this single case with very short follow-up. The incomplete immunologic work-up for PNP, lack of histologic confirmation of BO, and deviation from usual PNP therapeutic practice are important limitations that should be considered when interpreting this report.

## Data Availability

The raw data supporting the conclusions of this article will be made available by the authors, without undue reservation.
